# Haemostatic profile of children with nephrotic syndrome attending University of Nigeria Teaching Hospital Ituku-Ozalla, Nigeria

**DOI:** 10.1186/s12882-022-02894-5

**Published:** 2022-08-04

**Authors:** Chioma L. Odimegwu, Anthony N. Ikefuna, Henrietta U. Okafor, Theresa Nwagha, Agozie Ubesie, Josephat M. Chinawa

**Affiliations:** 1 Department of Paediatrics, College of Medicine, University of Nigeria Enugu Campus, PMB 40001, Enugu, Nigeria; 2 Department of Haematology and Blood Transfusion, College of Medicine, University of Nigeria Enugu Campus, Enugu, Nigeria

**Keywords:** Coagulation, Haemostasis, Nephrotic syndrome, Children, Nigeria

## Abstract

**Background:**

Haemostatic derangements are thought to be due to an imbalance between hepatic synthesis of pro-coagulants and urinary losses of anticoagulants.

**Objectives:**

This study evaluated the coagulation profile of Nigerian children with nephrotic syndrome and examined the relationship between coagulation variables, disease state and steroid responsiveness.

**Methods:**

A cross- sectional hospital based study on evaluation of coagulation profile of children with nephrotic syndrome compared with their age- and gender- matched controls.

**Results:**

The median fibrinogen level in subjects and controls was the same (2.9 g/L). Sixteen of 46 (35%) children with nephrotic syndrome had hyperfibrinogenaemia. The median fibrinogen level of children in remission was 2.3 g/L and differed significantly when compared with those of children in relapse (*p* = *0.001*). The median APTT of children with nephrotic syndrome was 45.0 s and differed significantly compared with those of controls (42.0 s) *(p value* = *0.02*). The median prothrombin time in children with and without nephrotic syndrome were 12.0 and 13.0 s respectively*, (p* = *0.004).* About 90% of children with nephrotic syndrome had INR within reference range. Thrombocytosis was found in 15% of children with nephrotic syndrome. The median platelet count in children with new disease was 432 × 10^3^cells/mm^3^ and differed significantly when compared with those of controls *(p* = *0.01*).

INR was significantly shorter in children with steroid resistant nephrotic syndrome (SRNS) (median 0.8 s; IQR 0.8 -0.9 s) compared with controls (median 1.0 s; IQR 1.0 -1.1 s) *(p* = *0.01)*. Steroid sensitivity was the strongest predictor of remission in children with nephrotic syndrome; steroid sensitive patients were 30 times more likely to be in remission than in relapse (OR 30.03; CI 2.01 – 448.04).

**Conclusion:**

This study shows that the haemostatic derangements in childhood nephrotic involve mostly fibrinogen, APTT, PT, INR and platelet counts. Antithrombin levels are largely unaffected. Variations in fibrinogen, APTT, PT and INR values may be due to the heterogeneous nature of the disease.

## Introduction

In nephrotic syndrome, there is increased permeability of the glomerular capillary wall leading to massive losses of protein in urine with resultant hypoalbuminaemia [1]. Apart from urinary loss of albumin, some anticoagulation factors namely antithrombin, protein C and protein S are also lost in urine of affected children. Consequently, there is diminished plasma anticoagulant factor levels, and increased predisposition to hypercoaguability of blood in the vascular system [1, 2].

Other predisposing factors to abnormal coagulation in children with nephrotic syndrome include increased platelet count [3], abnormal platelet function [3], hypovolaemia [4] and increased hepatic synthesis of pro-coagulants, namely Factors I, V, and VIII [1]. A combination of these factors increases the risk of the child with nephrotic syndrome towards a hypercoaguable state which may manifest clinically as thromboembolic events.

The risk of thromboembolism (TE) in children is significant, with at least one thromboembolic event occurring in approximately 9% of patients [5]. Studies in Bulgaria [6], Turkey [7] Canada [8] have reported an incidence of 2–5%, whereas another report in the US [9] was 9%.These figures, however are believed to be much higher in reality, since most thromboembolic events are clinically silent and are often incidentally discovered on CT scans [3]. To the best of my knowledge, there are no studies with documented reports on thromboembolic events in Nigerian children with nephrotic syndrome.

These thromboembolic complications have emerged as a major hazard of the nephrotic syndrome [4]. Renal vein thrombosis is particularly frequent in patients with membranous glomerulonephritis as documented by retrospective and prospective studies [4–10]. Most patients with renal venous thrombosis are asymptomatic, with only 10 percent presenting with symptoms: flank pain, gross haematuria, increased renal size, and loss of renal function [4]. Another hazard is pulmonary embolism, which is frequently clinically silent and is manifested clinically in only a minority of patients [4, 10–15].

The aim of the study therefore was to determine the coagulation indices (Fibrinogen, APTT, PT/INR, platelet count, AT) compared with their age- and gender- matched controls. The knowledge of these coagulation indices may be useful in predicting possible clinical outcome of the disease. It could also serve as a clinician’s guide to early and appropriate intervention while improving the overall management of children with this disorder.

## Methods

### Study area

The study was carried out at the University of Nigeria Teaching Hospital (UNTH) Ituku-Ozalla, Enugu state. Enugu state is in the South East geopolitical zone of Nigeria.

### Study population

These were children aged one to eighteen years with or without nephrotic syndrome. The subjects were recruited from the Paediatric Nephrology Clinic and General Paediatric Ward while age- and gender- matched controls were recruited from apparently healthy children attending the Children Outpatient Clinic on a follow-up visit.

Children aged 1 to 18 years diagnosed with nephrotic syndrome who gave their assent while their parents/care-givers gave their consent were enrolled in the study. The diagnosis of nephrotic syndrome was made based on the presence or history of generalized oedema, massive proteinuria with 3 + or 4 + of protein on urinalysis by dipstick [1, 2] urine protein-creatinine ratio greater than 2 mg/mg, serum albumin less than 25 g/L [1, 2] hypercholesterolaemia with serum cholesterol greater than 5.2 mmol/L [1, 2] and the histologic diagnosis from kidney biopsy where available.

Children aged 1 to 18 years without signs and symptoms of acute or chronic illness presenting to Children Outpatient (CHOP) clinic on a follow-up visit who gave their assent while their parents/caregivers gave their consent were recruited into the study. A urinalysis dipstick test for proteinuria was done for all control participants. Those with negative proteinuria were enrolled in the study. Those with positive proteinuria were referred to the Paediatric Nephrology clinic for further evaluation.

All participants with a history of bleeding diathesis/clotting disorder or on medications that could affect/influence these e.g. Heparin/Warfarin, those on non-steroidal anti-inflammatory drugs (NSAIDs), steroids and those with other chronic clinical conditions (e.g. Sickle cell anemia, Asthma, Epilepsy, Diabetes, End stage renal disease ESRD) were excluded from the study.

### Study design

A cross- sectional hospital based study on evaluation of coagulation profile of children with nephrotic syndrome compared with their age- and gender- matched controls.

### Sample size estimation

The minimum sample size for this study was calculated using the formula for comparison [16].

$$\mathrm{n}=\frac{{2\left({\mathrm{Z}}_{a}+{\mathrm{Z}}_{1-\upbeta }\right)}^{2}{\upsigma }^{2}}{{\Delta }^{2}}$$

Using the above formula, the minimum sample size is 44.

5% attrition rate was considered [17, 18] and this brought the final value to 46.

Thus, 46 children with nephrotic syndrome and 46 apparently healthy controls were enrolled in the study.

### Ethical approval and consent

The approval of the Health Research Ethics Committee of the University of Nigeria Teaching Hospital, Enugu was obtained. Patients and parents or caregivers were duly informed in detail about the purpose of the study, the specimen for the study and its method of collection. A written consent was obtained from parents or caregivers of all study participants while an assent was obtained in participants aged 7 years and older.

### Sampling technique

A non-probability (convenient) sampling method was employed in the study. Children with nephrotic syndrome who met the inclusion criteria were consecutively enrolled in the study until the desired sample size was achieved. Children without nephrotic syndrome who met the inclusion criteria were age- and gender- matched with those with nephrotic syndrome and also enrolled by convenient sampling technique.

### Study duration

The study was carried out over a six-month period (December 2017 to May 2018).

## Results

### Characteristics of the study population

The study was carried out over a six-month period (December 2017 to May 2018). A total of 46 children with nephrotic syndrome and an equal number of apparently healthy age- and gender-matched controls who met the inclusion criteria were enrolled into the study. The age range of the study participants was 2 -18 years. There were 28 males and 18 females, with a male to female ratio of 1:0.6. Descriptive characteristics of study participants are shown in Table 1.

### Coagulation profile of study participants

#### Fibrinogen

The range of fibrinogen among children with nephrotic syndrome was 1.1 to 7.2 g/L (median 2.9 g/L) while that of children without nephrotic syndrome was 1.4 to 4.5 g/L (median 2.9 g/L). Sixteen (35%) of 46 children with nephrotic syndrome had hyperfibrinogenaemia. The difference in the median fibrinogen concentrations of the subjects and controls was not statistically significant (*p* = 0.568; Table 2).

**Table 1:** Socio-demographic characteristics of the study participants

	Children with Nephrotic Syndrome (Subjects)	Children without Nephrotic syndrome (Controls)	
Sample size (N)	46	46	
Sex (n %)			
Female	18 (38)	18 (38)	
Male	28 (61)	28 (61)	
Age (years)			
Median	11.0	11.0	
IQR	8.0 – 16.0	8.0 – 15.25	
95% CI	10.2 – 12.9	10.1 – 12.8	

*SD* Standard deviation, *CI* Confidence Interval, *IQR* Inter-quartile range

**Table 2:** Coagulation profile of children with and without nephrotic syndrome

Variable (unit) (Ref range)	Children with nephrotic syndrome	Children without nephrotic syndrome	*p value*
**Fibrinogen (g/L) (1.5–3.5)**			
Median	2.9	2.9	***0.568***
IQR	2.1 -4.9	2.3 – 3.4	
95% CI of Mean	2.9 – 3.9	2.7 – 3.2	
**APTT (s) (36–50)**			
Median	45.0	42.0	0.021*
IQR	39.0 -57.0	38.0—45.3	
95% CI of Mean	45.48 – 53.12	40.7 – 45.9	
**PT (s) (11–16)**			
Median	12.0	13.0	0.004*
IQR	11.0 – 13.0	12.0 – 14.0	
95% CI of Mean	11.2 – 14.3	12.7 – 14.2	
**INR (0.8–1.2)**			
Median	0.9	1.0	0.009*
IQR	0.8 – 1.0	1.0 -1.1	
95% CI of Mean	0.9 – 1.1	1.0 – 1.1	
**Platelet Count (x10**^**3**^**cells/mm**^**3**^**)** **(150–400)**			
Median	258	235	***0.310***
IQR	181 – 339	197 – 278	
95% CI of Mean	240 – 308	225 – 267	

*SD* Standard deviation, *IQR* Inter-quartile range, *APTT* Activated partial thromboplastin time, *PT* Prothrombin time, *INR* International normalized ratio, *AT* Antithrombin, *CI* Confidence interval

^*^ Statistically significant (*p* < 0.05)

#### Antithrombin (AT)

The median antithrombin values were 142% and 131% in children with and without nephrotic syndrome respectively. The difference in the median ATIII of the subjects and controls was not statistically significant (*p* = 0.134) Table 2.

#### Activated partial thromboplastin time (APTT)

The range of APTT in children with and without nephrotic syndrome was 35 to 75 s (median 45 s) and 35 to 50 s (median 42 s) respectively. Fifteen (33%) of 46 children with nephrotic syndrome had prolonged APTT. The difference in the median APTT values of subjects and controls was statistically significant (*p* value = 0.02) as shown in Table 2.

#### Prothrombin time (PT)

The range of prothrombin time in children with and without nephrotic syndrome was 8.0 to 41.0 s and 9.9 to 17.0 s respectively. The median prothrombin timewas 12 s (IQR 11.0 -13.0 s) and 13 s (IQR 12.0 -14.0) in subjects and controls respectively (*p* = 0.004). Thirty-nine (85%) out of 46 children with nephrotic syndrome had normal prothrombin time Table 2.

#### International normalized ratio (INR)

5 (11%) of 46 children with nephrotic syndrome had INR above reference range. The median INR in subjects and controls were 0.9 s (IQR 0.8 -1.0 s) and 1.0 s (1.0 – 1.1 s) respectively. INR differed significantly in children with nephrotic syndrome compared with controls (*p* = 0.009; Table 2).

#### Platelet count

In children with nephrotic syndrome, platelet counts ranged from 109 to 542 × 10^3^cells/mm^3^, and 155 to 384 × 10^3^cells/mm.^3^ in children without nephrotic syndrome, (*p* = 0.310). Seven (15%) of 46 subjects had platelet count above reference range Table 2.

### Coagulation profile of children with nephrotic syndrome in various disease states

#### Fibrinogen

The median plasma fibrinogen levels were 4.9, 4.1, 2.3 and 3.0 g/L in onset, relapse, remission and controls respectively. Four out of 7 (57%) children with onset and 10 out of 19 (52%) children in relapse had fibrinogen levels above the upper limit of normal. Nineteen out of 20 (95%) of children in remission had fibrinogen levels within reference range. There was statistically significant difference in plasma fibrinogen levels of those in relapse compared to those in remission (*p* = 0.001). The difference in median fibrinogen of children in remission compared to controls was also significant (*p* = 0.001; Table 3).

**Table 3:** Coagulation profile in various disease state and control

	New Disease	Relapse	Remission	Control	*Group p value*	*ND vs Rel*	*ND vs Rem*	*ND vs Con*	*Rel. Vs Rem*	*Rel. Vs Con*	*Rem vs Con*
**Sample size (N)**	7	19	20	46							
**Fibrinogen (g/l) (1.5 – 3.5)**											
Median											
Median	4.9	4.1	2.3	3.0	0.001*	** > *0.05***	** > *0.05***	** > *0.05***	0.001*	** > *0.05***	0.001*
IQR	0.9 -6.2	2.3 – 5.5	1.9 – 2.6	2.4 – 4.0							
**AT (%) (80–140)**											
Median	141.0	142.0	143.5	131.0	***0.334***	** > *0.05***	** > *0.05***	** > *0.05***	** > *0.05***	** > *0.05***	** > *0.05***
IQR	118.0 – 148.0	128.0 – 152.0	130.5 -149.0	125.0 -146.0							
**APTT (s) (36 -50)**											
Median	43.0	46.0	39.8 -50.0	42.0	***0.148***	** > *0.05***	** > *0.05***	** > *0.05***	** > *0.05***	** > *0.05***	** > *0.05***
IQR	38.0 – 69.5	39.8 -68.3		38.0 -45.3							

*ND* New Disease, *Con* Control, *Rel* Relapse, *Rem* Remission, *SD* Standard deviation, *IQR* Inter-quartile range, *Plt* Platelet, *APTT* Activated partial thromboplastin time, *PT* Prothrombin time, *INR* International normalized ratio, *AT* Antithrombin, *Fib* Fibrinogen

^*^ Statistically significant (*p* < 0.05)

#### Antithrombin (AT)

The median values of AT were 141%, 142%, 144% and 131% in onset, relapse, remission and controls respectively. AT values did not differ significantly between various disease states when compared in pairs (*p* > 0.05).

#### Activated partial thromboplastin time (APTT)

The median APTT values were 43 s, 46 s, 46 s and 42 s in onset, relapse, remission and controls respectively. Three out of 7 (42.8%) children with onset and 7 out of 19 (36.8%) children in relapse had prolonged APTT while 4 of 20 (20%) children in remission had prolonged APTT. Median APTT values were longer in children with nephrotic syndrome in various disease states compared with controls, although no significant difference was observed (Group *p* = 0.148) Table 3. 

#### Prothrombin time (PT)

The prothrombin time was the same in onset, relapse and remission. The difference between PT in various disease states compared with controls was significant (*p* = 0.024; Table 4). Seventy one percent of children with onset, 95% (19 out of 20) of children in remission and 95% (18 out of 19) of children in relapse had prothrombin time within normal reference range. Children with nephrotic syndrome had relatively shorter PT compared with controls (= 0.024; Table 4).

**Table 4:** Coagulation profile in various disease state and control

	New Disease	Relapse	Remission	Control	*Group* *p value*	*ND* *vs Rel*	*ND* *vs Rem*	*ND* *vs Con*	*Rel* *Vs Rem*	*Rel* *Vs Con*	*Rem vs* *Con*
**Sample size (N)**	7	19	20	46							
**PT (s) (11 -16)**											
Median	12.0	12.0	12.0	13.0	0.024*	** > *0.05***	** > *0.05***	** > *0.05***	** > *0.05***	** > *0.05***	** > *0.05***
IQR	12.0 – 15.0	10.0 -13.0	11.0 – 13.0	12.0 – 14.0							
**INR (0.8 -1.2)**											
Median	1.1	0.9	0.9	1.0	0.009*	** > *0.05***	** > *0.05***	** > *0.05***	** > *0.05***	** > *0.05***	** > *0.05***
IQR	0.9 – 1.2	0.8 – 1.0	0.8 – 1.0	0.9 -1.1							
**Platelet Count** ( **x10**^**3**^**cells/mm**^**3**^) **(150 -400)**											
Median	432	259	233	235	0.047*	** > *0.05***	** > *0.05***	0.01*	** > *0.05***	** > *0.05***	** > *0.05***
IQR	268—512	165—301	181—274	197—278							

*ND* New Disease, *Con* Control, *Rel* Relapse, *Rem* Remission, *SD* Standard deviation, *IQR* Inter-quartile range, *Plt* Platelet, *APTT* Activated partial thromboplastin time, *PT* Prothrombin time, *INR* International normalized ratio, *AT* Antithrombin, *Fib* Fibrinogen

^*^ Statistically significant (*p* < 0.05)

#### International normalized ratio (INR):

5 (71.4%) of 7 children with onset had INR within reference range while 4(21%) of 19 children in relapse had INR below reference range. The median INR values in various disease states and controls.

#### Platelet count

The median platelet counts in children with onset, relapse, remission and controls were 432 × 10^3^cells/mm^3^ (IQR 268 -512 × 10^3^ cells/mm^3^), 259 × 10^3^cells/mm^3^ (IQR 165 -301 × 10^3^cells/mm^3^), 233 × 10^3^cell/mm^3^ (181 -274 × 10^3^cells/mm^3^) and 235 (197 -278 × 10^3^cells/mm^3^) respectively. Seventeen of 19 (89%) of children in relapse and 19 of 20 (95%) children in remission had platelet counts within reference range. Four of 7 (57%) subjects with onset had elevated platelet counts. There was statistically significant difference in platelet counts of children with onset compared with children without nephrotic syndrome (*p* = 0.01; Table 4).

### Coagulation profile in Steroid Sensitive Nephrotic Syndrome (SSNS) and Steroid Resistant Nephrotic Syndrome (SRNS)

In this study, children with nephrotic syndrome who had been on steroid therapy were classified as either steroid sensitive or steroid resistant based on their steroid responsiveness. In both categories there were children who were either in remission or in relapse. Coagulation profile in steroid sensitive and steroid resistant nephrotic syndrome is as shown in Table 5.

**Table 5:** Coagulation profile steroid-sensitive and steroid resistant nephrotic syndrome and controls

**Sample size (N)** **Variable Fib(g/L)**	SSNS 24	SRNS 15	CS 46	Group *p* value	SS vs SR	SS vs CS	SR vs CS
Median	2.4	3.7	3	***0.051***^**+**^	** > *0.050***	** > *0.050***	** > *0.050***
IQR	2.0 – 3.3	2.0—5.5	2.4 – 4.0				
**AT (%)**							
Median	142.5	144	131	***0.187***	** > *0.050***	** > *0.050***	** > *0.050***
IQR	128.0–149.0	128.0 -152.0	125—146				
**APTT (s)**							
Median	49	43	42	0.025*	** > *0.050***	0.010*	** > *0.050***
IQR	40.3 – 64.3	39.0—50.0	38.0—45.3				
**PT (s)**							
Median	12	11	13	0.010*	** > *0.050***	** > *0.050***	0.010*
IQR	11.0 – 13.0	10.0 -12.0	12.0 -14.0				
**INR**							
Median	0.9	0.8	1	0.004*	** > *0.050***	** > *0.050***	0.001*
IQR	0.9 -1.0	0.8—0.9	1.0—1.1				
**Plt Count**							
(**x10**^**3**^**cells** **/mm**^**3**^) Median	238	257	235	***0.781***	** > *0.050***	** > *0.050***	** > *0.050***
IQR	183 -328	152 -180	197 -277				

*SR* Steroid resistant, *SS* Steroid sensitive, *CS* Controls, *vs* Versus, *SSNS* Steroid sensitive nephrotic syndrome, *SRNS* Steroid resistant nephrotic syndrome, *SD* Standard deviation, *IQR* Inter-quartile range, *Plt* Platelet, *APTT* Activated partial thromboplastin time, *PT* Prothrombin time, *INR* International normalized ratio, *AT* Antithrombin, *Fib* Fibrinogen

^*^ Statistically significant (*p* < 0.05)

^+^ Group *P* value = trend towards statistical significance

#### Fibrinogen

The median fibrinogen concentration in steroid sensitive nephrotic syndrome (SSNS) and steroid resistant nephrotic syndrome (SRNS) were 2.4 g/L (IQR 2.0 -3.3 g/L) and 3.7 g/L (IQR 2.0 –5.5 g/L). Eight out of 15 (53.3%) children with steroid resistant nephrotic syndrome (SRNS) had elevated fibrinogen while 5 out of 24 (20.8%) children with steroid sensitive nephrotic syndrome (SSNS) had elevated fibrinogen levels. There was no significant difference between fibrinogen concentration in SRNS and SSNS (*p* > 0.05), however there was a trend towards statistical significance (Group *p* value = 0.051) as shown in Table 5.

#### Antithrombin (AT)

The median anithrombin (AT) levels in steroid sensitive nephrotic syndrome (SSNS) and steroid resistant nephrotic syndrome (SRNS) were 142.5% (IQR 128.0 – 149.0%) and 144.0% (IQR 128.0 – 152.0) respectively (*p* > 0.05; Group *p* value = 0.187).

#### Activated partial thromboplastin time (APTT)

Eight of 24 (33%) of children with SSNS had prolonged APTT while 5 of 15 (33%) children with SRNS had prolonged APTT. The median APTT value was 49 s (IQR 40.3 s – 64.3 s) and 43 s (IQR 39.0 -50.0) in SSNS and SRNS respectively (*p* = 0.346) as shown in Table 6. APTT in SSNS did not differ significantly compared with SRNS.

#### Prothrombin time (PT)

14 (93.3%) of 15 children with SRNS had PT within normal reference range while 21(87.5%) of 24 children with SSNS had PT within normal reference range. The median prothrombin time (PT) was 12.0 s and 11.0 s in SSNS and SRNS respectively (*p* = 0.175; Table 5) and 11.0 and 13.0 s in SRNS and controls respectively. There was statistically significant difference in PT between SRNS and controls (*p* = 0.01, Table 5).

#### International Normalized Ratio (INR)

The median INR was 0.9 s (IQR 0.9 -1.0 s) and 0.8 s (IQR 0.8—0.9 s) in SSNS and SRNS respectively (*p* > 0.05). Median INR was 0.8 and 1.0 s in SRNS and controls respectively. There was no statistically significant difference in INR between SRNS and SSNS (*p* > 0.05).

#### Platelet count

The median platelet count in children with SSNS and SRNS was 238 × 10^3^cells/mm (IQR 183 -328 × 10^3^cells/mm^3^) and 257 × 10^3^cells/mm3 (IQR 152 -180 × 10^3^cells/mm^3^) respectively, (*p* > 0.05) as shown in Table 6. All children with SRNS had platelet counts within reference range while 12.5% (3 of 24) of children with SSNS had platelet counts above reference range.

### Predicting relationship between coagulation variables, steroid response status and disease state

Coagulation variables were regressed on steroid response status using logistic regression (Hierarchical Forward Cluster method). Steroid response status is a categorical variable that has only two outcomes: steroid sensitive or steroid resistant. Results showed that none of the coagulation parameters were predictors of steroid sensitivity in childhood nephrotic syndrome; fibrinogen (OR 0.58; CI 0.33—1.02) and prothrombin time (OR 1.38; CI 0.91—2.07; Table 6).

**Table 6:** Logistic regression of coagulation variables on steroid response

	Regression	Odds	Lower 95%	Upper 95%	
Independent	Coefficient	Ratio	Confidence	Confidence	
Variable	b(i)	Exp(b(i))	Limit	Limit	
Fibrinogen	-0.54238	0.58	0.33	1.02	
PT	0.31857	1.38	0.91	2.07	

Estimated Logistic Regression Model(s)

Model For Steroid Sensitive Nephrotic Syndrome

2.55–0.50*Fib + 3.69*INR -0.23*BMI

Another multiple logistic regression was performed on coagulation variables using disease state as the outcome variable. Disease state is a categorical variable with three possible outcomes: new disease, relapse and remission. The results showed that steroid sensitivity was a strong predictor of remission in children with nephrotic syndrome (OR 30, CI 2.01—448.04; Table 7).

**Table 7:** Logistic regression of coagulation variables and steroid response pattern on disease state

	Regression	Odds	Lower 95%	Upper 95%
Independent	Coefficient	Ratio	Confidence	Confidence
Variable	b(i)	Exp(b(i))	Limit	Limit
Fibrinogen	-2.118	0.12	0.03	0.56
Steroid Sensitive	3.402	30.03	2.01	448.04

Estimated Logistic Regression Model(s)

Model For Remission in Nephrotic Syndrome 9.03–2.18*Fib + 3.40*2 -0.10

### Association between coagulation variables and steroid response status

When values of coagulation variables where trichotomised into high, normal and low categories, strength of association of coagulation variables with steroid response status (sensitive and resistant) were tested for using Chi-square. In Table 8 only fibrinogen and INR showed a significant association with steroid response status. Normal fibrinogen (1.5 -3.5 g/L) is significantly associated with steroid sensitivity (χ^2^ value = 9.798; *p* = 0.008). Elevated fibrinogen (> 3.5 g/L) is significantly associated with steroid resistant nephrotic syndrome (χ^2^ value = 9.798; *p* = 0.008). A normal INR (0.8 -1.2) is significantly associated with steroid sensitive nephrotic syndrome, while a lower INR (< 0.8) is significantly associated steroid resistant nephrotic syndrome (χ^2^ value = 6.715; *p* = 0.035). The other variables (AT, APTT, PT and platelet count) had no significant association with steroid response status (*p* > 0.05), Table 8.

**Table 8:** Association between coagulation variables and steroid response status

Variable	No of cases			
	Steroid Sensitive Nephrotic syndrome	Steroid Resistant Nephrotic syndrome	Chi Square χ^2^	*p* value
High Fib (> 3.5 g/L)	1	8	9.798	0.008*
Normal Fib ( 1.5 -3.5 g/L)	22	6		
Low Fib (< 1.5 g/L)	1	1		
High AT (> 144%)	11	8	0.303	0.859
Normal AT (80 -144%)	11	6		
Low AT-III (< 80%)	2	0		
High APTT (> 50 s)	7	3	0.037	0.982
Normal APTT ( 36 -50 s)	15	11		
Low APTT (< 36 s)	2	1		
High PT (> 16 s)	1	0	0.335	0.846
Normal PT (11 -16 s)	17	11		
Low PT (< 11 s)	6	3		
High INR (> 1.2)	5	2	6.715	0.035*
Normal INR (0.8 -1.2)	19	7		
Low INR (< 0.8)	0	6		
High PLT (> 400 × 10^3^ cells/mm^3^	2	0	1.459	0.482
Normal PLT (150 – 400 × 10^3^ cells/mm^3^)	18	12		
Low PLT (< 150 × 10^3^ cells/mm^3^)	4	3		

*Key*: *Plt* Platelet, *APTT* Activated partial thromboplastin time, *PT* Prothrombin time, *INR* International normalized ratio, *AT* Antithrombin, *Fib* Fibrinogen

^*^ Statistically significant *p* < 0.05

## Discussion

The median fibrinogen concentration in children with nephrotic syndrome was 2.9 g/L. This was comparable to the median fibrinogen concentration among the controls (*p* = 0.568). The median fibrinogen concentration of both subjects and controls were within the reference range. A possible explanation for the lack of significant difference between the median fibrinogen of subjects and controls may be the heterogeneous nature of the nephrotic syndrome group i.e. a mixture of subjects with active disease and those in remission. However, when considered in various disease states, median fibrinogen levels differed significantly between children in relapse and those in remission (*p* = 0.001).This finding agrees with publications by Elidrissy et al., [19] Mugeiren et al., [20] Citak et al. [7]and Rani et al. [21] These authors found elevated fibrinogen levels in children with active disease (onset or relapse). Hyperfibrinogenemia occurs due to increased hepatic synthesis in response to glomerular losses of albumin in nephrotic syndrome and has been associated with increased risk of hypercoagulability and thromboembolism. [7, 22–26] Conversely, about 95% of children in remission from this study had fibrinogen levels within the reference range. This was similar to reports by several authors [22–26] and underscores an inverse relationship between serum fibrinogen and albumin levels [7].

The median antithrombin values were 142% and 131% in children with and without nephrotic syndrome respectively and both were within reference range. There was no significant difference in anithrombin levels among children with nephrotic syndrome compared with apparently healthy controls. Additionally, when considered in various disease states, median antithrombin levels were all within reference range and did not differ significantly compared within the disease states and with controls. The finding of normal antithrombin levels in children with nephrotic syndrome in this study suggests that the quantitative levels of antithrombin are unaffected in childhood nephrotic syndrome.This is further strengthened by a recent study published in 2015 by Kerlin et al. [9] which observed that plasma antithrombin concentration was not significantly altered by proteinuria in nephrotic syndrome, rather antithrombin activity was significantly reduced. The authors concluded that the well-known antithrombin (AT) deficiency in active nephrotic syndrome might rather have been caused by a qualitative defect in enzymatic activity rather than a reduction in quantitative levels of AT. Contrary to the finding of this study, many authors have previously reported low AT levels in children with nephrotic syndrome, especially those with active disease; al-Mugerin et al. [22] and Ueda et al. [26] both reported low AT levels in children with relapsed state of nephrotic syndrome while Wygledowska et al. [27] and Anand et al. [28] reported low AT in children with onset. Mittal et al. [29] found low AT levels in NS patients with onset and in relapse, although the values were within the lower limit of the normal and did not differ significantly when compared with those of children in remission. This reduction in AT level was believed to be due to urinary losses of antithrombin, a 58kilodaltons (kD) intermediate molecular mass protein alongside albumin.Earlier assays of AT measured only enzymatic activity, but newer immunoassays now measure AT concentration. As such, the antithrombin human in vitro Enzyme-Linked Immunosorbent Asssay (ELISA) kit employed in this study measured the quantititative levels of AT in plasma and found that children with nephrotic syndrome in our environment had AT levels within reference range.

The median activated partial thromboplastin time (APTT) in children with and without nephrotic syndrome was 45.0 s and 42.0 s respectively. There was significant difference in median APTT values between subjects and controls. Also, median values of APTT in onset and relapse were prolonged (outside reference range) and higher in active disease compared with those of children in remission. Similar observations have been made by Rani et al. [21] and Anand et al. [30] Rani and colleagues reported significantly prolonged APTT in relapse compared with those of children in remission and to controls [21]. The mechanism of prolonged APTT in active nephrotic syndrome is mostly linked to loss of pro-coagulant proteins in urine. Haemostatic proteins up to 80 kDaltons, including Factors II, VI, IX, XI and XII are reportedly lost in urine in NS [30, 31]. Secondly, prolonged APTT in active disease may also be due to increased AT activity and increased fibrinolytic state [30]. On the contrary however, Mittal et al. [29] in their 2013 study reported that APTT was shortened in relapse but became prolonged in remission. The authors postulated that the shortened APTT seen in relapse may be due to an increase in hepatic synthesis of pro-coagulants in response to urinary losses of albumin and antithrombin [29]. Although studies have shown that the interplay of pro-coagulant and anticoagulant factors ultimately describe the coagulation derangements seen in nephrotic syndrome [21, 29, 31–33] the rate and synthesis of these factors are not clearly elucidated [33, 34]. Patients with nephrotic syndrome tend to suffer from a spectrum of haemostatic disorders, ranging from an increased incidence of thromboembolism to an increased bleeding tendency [34, 35]. The median prothrombin time in subjects and controls was 12 s and 13 s respectively. Although prothrombin time (PT) was significantly shorter in children with nephrotic syndrome compared with controls, both subjects and controls had median PT within reference range. There was no significant difference observed in PT in the various clinical states of disease when compared with each other. Similar results were reported by some authors who found that children with nephrotic syndrome had normal prothrombin time and no significant differences in PT values when compared between disease pairs such as remission and relapse [21, 29, 30] and onset and control [29] Although the authors offered no explanation for this finding, it is plausible to think that the coagulation factors involved in the extrinsic pathway i.e. Factors III and VII are not lost in urine. This is despite the fact that Factor VII is a low molecular weight protein of 50kD [29]. It is therefore possible that the extrinsic pathway is unaffected by the pathophysiologic changes that occur in nephrotic syndrome.

About 90% of children with nephrotic syndrome had INR within reference range. The median INR in subjects and controls was 0.9 s and 1.0 s respectively. INR differed significantly in children with nephrotic syndrome compared with controls. However, no statistically significant differences were observedwithin various disease states and controls. This finding is similar to those of prothrombin time as reported by several authors [21, 29, 30] and may be explained by the direct positive correlation between PT and INR.

The median platelet counts did not differ significantly between subjects and controls. About 90% of children in relapse and 95% of children in remission in my study had platelet counts within reference range. There was no significant difference in platelet counts of children in relapse, compared with those of children in remission or with controls.However, thrombocytosis was observed in 57% of subjects with onset. Platelets are known to play a crucial role in the pathogenesis of thrombotic changes in NS [3, 31, 34]. Although the exact mechanism of elevated platelet count in nephrotic syndrome is unknown, it is assumed that biochemical disorders associated with hypoalbuminaemia lead to increased platelet aggregation and are responsible for the elevated platelet counts [3, 34]. Hypoalbuminaemia correlates negatively with platelet count, as such platelet counts are expected to return to normal as the patient goes into remission [3]. Similar findings have been reported by Anand and colleagues [30] who observed thrombocytosis in children with onset, which differed significantly when compared with the controls.Contrary to my findings, some studies reported statistically significant higher platelet counts in children in remission compared to controls [3, 30]^.^ A closer look at the study by Wasilewska*et al* [3] revealed that the researchers in a longitudinal study commenced steroids in children with active disease and achieved remission within two weeks. The samples for platelet count were then collected in the early phase of remission, as such; platelet counts were yet to return to normal. The differences in the results may therefore be accounted for by the phase of treatment at which blood samples were collected and analysed.

The median fibrinogen concentrations in SSNS and SRNS were 2.4 and 3.7 g/L respectively. Although fibrinogen levels were elevated above reference range in children with SRNS, no significant difference was observed when compared with children with SSNS. However a trend towards statistical significance was observed. Although it is believed that trends signify a ‘near significant’ or ‘almost/approaching statistical significant’ result when the *p* value is close to < 0.05 [35], recent publications however do not seem to support these thoughts [36, 37]. In a 2014 study by Wood et al. [36] the authors observed that the *p* value by no means became smaller even with the addition of quite a substantial proportion of extra data. The authors therefore concluded that describing near significant *p* values as “trends towards significance” was as inappropriate as it was misleading [36]. A similar observation was also reported by Gibbs [37] et al.in 2015 wherein the authors also concluded that the use of trend to describe ‘almost significant’ differences was an error both in word usage and statistical inference.

No other significant differences were observed in the median values of AT, APTT, PT, INR and platelet count between steroid sensitive nephrotic syndrome and steroid resistant nephrotic syndrome. The reason for this could not be explained.

Children with steroid resistant nephrotic syndrome had significantly shorter prothrombin time compared with controls. INR was significantly shorter in children with steroid resistant nephrotic syndrome compared to controls. This suggests that prolonged steroid therapy may shorten PT and reduce INR. Children with SSNS had significantly longer APTT compared with controls. However, when compared with SRNS, APTT was relatively longer in SSNS, although the difference was not statistically significant. This also suggests that prolonged steroid therapy may shorten APTT as seen in SRNS (median 43 s) which was comparable to those of controls (median 42 s). Several authors have reported the effect of steroid on coagulation variables in nephrotic syndrome [38–40] however, not much has been documented on coagulation variables in various steroid response states. In a study by Anand [30] et al.in 1996 among Indian children with nephrotic syndrome, the researchers found that children with steroid resistant nephrotic syndrome (SRNS) had normal APTT levels similar to findings in this study. The authors also reported that the AT levels was significantly higher in SRNS compared to controls. This was also similar to the findings in this study, although the difference observed was not significant. Contrary to the finding in this study, Anand and co-authors reported that PT in SRNS was slightly prolonged compared to controls, while platelet count in SRNS was markedly elevated above reference range (522.4 ± 61) compared with controls (117.5 ± 37.4) [30]. The authors postulated that fluctuations in coagulation variables in childhood nephrotic syndrome are determined by response to steroid therapy and not the renal pathology per se. The limitation of Anand’s study is that children with SSNS were not included in their study [30].

To further establish the relationship between steroid response and coagulation variables, a Spearman correlation was done. This study found that in children with steroid sensitive nephrotic syndrome (SSNS), platelet count had a moderate correlation with antithrombin (Rho = 0.34, *p* = 0.10). However, antithrombin had a weak but inverse correlation with INR. In steroid resistant nephrotic syndrome, platelet count had a moderate correlation with fibrinogen. However, antithrombin had a good but inverse correlation with prothrombin time. Using a logistic regression, it was found that steroid sensitive children were 30 times more likely to be in remission than in relapse. Further studies on coagulation variables in steroid response status will be needed to strengthen these findings.

In this study, hyperfibrinogenaemia (> 3.5 g/L) was significantly associated with steroid resistant nephrotic syndrome. Several reports have shown that hyperfibrinogenaemia is associated with increased risk of thromboembolic complications [30]. Anand [30] et al. found hyperfibrinogenemia in 100% of children who had thromboembolic complications in their study. Lilova [6] et al. reported a higher incidence of thromboembolic complication (TEC) in children with steroid resistant nephrotic syndrome (SRNS) compared with steroid sensitive nephrotic syndrome (SSNS). Similarly, Eldrissy [19] et al. reported hyperfibrinogenaemia in children with steroid resistant nephrotic syndrome. It is therefore evident that the higher incidence of TEC in SRNS is related to hyperfibrinogenaemia seen in these children.

Also in this study, a lower INR (< 0.8) was significantly associated with steroid resistant nephrotic syndrome. A lower INR is associated with a hypercoagulable state and will also explain the increased incidence of thromboembolic complications seen in children with SRNS as reported by Livola [6] et al. and Eldrissy [19] et al.

The other variables (AT, APTT, PT and platelet count) had no significant association with steroid response status (*p* > 0.05).

## Conclusion

This study shows that the haemostatic derangements in childhood nephrotic involve mostly fibrinogen, APTT, PT, INR and platelet counts. Antithrombin levels are largely unaffected. Variations in fibrinogen, APTT, PT and INR values may be due to the heterogeneous nature of the disease.

### Recommendation

It is expedient from the study to recommend that plasma fibrinogen concentration should be monitored in all children with nephrotic syndrome. Furthermore, activated partial thromboplastin time, prothrombin time, platelet and INR should be measured in children with nephrotic syndrome, especially in those with onset and in relapse. A repeat platelet count should be done in late remission.

### Limitations

The study was cross-sectional in design and did not allow for follow up on subjects. Sample sizes in various disease states were relatively small. Functional assays such as enzymatic activity of antithrombin and platelet aggregation tests were not performed due to cost. There were limited numbers of assays on procoagulant factors due to cost constraints.

## Data Availability

Data are however available from the authors upon reasonable request and with permission of the corresponding Author.

## Abbreviations

AA: Arachidonic acid
ADP: Adenosine diphosphate
APTT: Activated partial thromboplastin time
AT: Antithrombin (also known as Antithrombin III)
FSGS: Focal segmental glomerulosclerosis
INR: International normalized ratio
INS: Idiopathic nephrotic syndrome
IQR: Inter-quartile range
MCD: Minimal change disease
MesPGN: Mesangial proliferative glomerulonephritis
MN: Membranous nephropathy
MPGN: Membranoproliferative glomerulonephritis;
NS: Nephrotic syndrome
PC: Protein C
PE: Pulmonary embolism
PLT: Platelet
PS: Protein S
PT: Prothrombin time
QMN: Quartan malaria nephropathy
SD: Standard deviation
SRNS: Steroid resistant nephrotic syndrome
SSNS: Steroid sensitive nephrotic syndrome
TE: Thromboembolism
TEC: Thromboembolic complications
TPL: Tissue phospholipids
TT: Thrombin time
TXA2: Thromboxane A2
UTI: Urinary tract infection
VTE: Venous thromboembolism
vWF: Von Willibrand factor
